# Mathematical Modeling and Numerical Simulation of Atherosclerosis Based on a Novel Surgeon’s View

**DOI:** 10.1007/s11831-021-09623-5

**Published:** 2021-07-08

**Authors:** Meisam Soleimani, Axel Haverich, Peter Wriggers

**Affiliations:** 1 Institute of Continuum Mechanics, Leibniz Universität Hannover, Hannover, Germany; 2 Department of Cardiothoracic, Transplantation, and Vascular Surgery, Hannover Medical School (MHH), Hannover, Germany

## Abstract

This paper deals with the mathematical modeling of atherosclerosis based on a novel hypothesis proposed by a surgeon, Prof. Dr. Axel Haverich (Circulation 135(3):205–207, 2017). Atherosclerosis is referred as the thickening of the artery walls. Currently, there are two schools of thoughts for explaining the root of such phenomenon: thickening due to substance deposition and thickening as a result of inflammatory overgrowth. The hypothesis favored here is the second paradigm stating that the atherosclerosis is nothing else than the inflammatory response of of the wall tissues as a result of disruption in wall nourishment. It is known that a network of capillaries called vasa vasorum (VV) accounts for the nourishment of the wall in addition to the natural diffusion of nutrient from the blood passing through the lumen. Disruption of nutrient flow to the wall tissues may take place due to the occlusion of vasa vasorums with viruses, bacteria and very fine dust particles such as air pollutants referred to as PM 2.5. They can enter the body through the respiratory system at the first place and then reach the circulatory system. Hence in the new hypothesis, the root of atherosclerotic vessel is perceived as the malfunction of microvessels that nourish the vessel. A large number of clinical observation support this hypothesis. Recently and highly related to this work, and after the COVID-19 pandemic, one of the most prevalent disease in the lungs are attributed to the atherosclerotic pulmonary arteries, see Boyle and Haverich (Eur J Cardio Thorac Surg 58(6):1109–1110, 2020). In this work, a general framework is developed based on a multiphysics mathematical model to capture the wall deformation, nutrient availability and the inflammatory response. For the mechanical response an anisotropic constitutive relation is invoked in order to account for the presence of collagen fibers in the artery wall. A diffusion–reaction equation governs the transport of the nutrient within the wall. The inflammation (overgrowth) is described using a phase-field type equation with a double well potential which captures a sharp interface between two regions of the tissues, namely the healthy and the overgrowing part. The kinematics of the growth is treated by classical multiplicative decomposition of the gradient deformation. The inflammation is represented by means of a phase-field variable. A novel driving mechanism for the phase field is proposed for modeling the progression of the pathology. The model is 3D and fully based on the continuum description of the problem. The numerical implementation is carried out using FEM. Predictions of the model are compared with the clinical observations. The versatility and applicability of the model and the numerical tool allow.

## Introduction

Discovering the mystery of plaque formation (atherosclerosis) in the arteries has always been a fascinating subject among the medical researchers since long time ago. In 1852, Rokitansky opined that an excessive accretion and deposition of a thrombotic layer from the blood stream on the intima caused atherosclerosis. In 1913, Anitschkow’s experiments conducted on aorta of rabbits have demonstrated a relation between elevated cholesterol level and lipid lesions. This led to intensive studies on relation of cholesterol metabolism to atherosclerosis [16]. Schwartz and team emphasize initial influx and accumulation of lipoprotein (LDL), and recruitment of monocytes as the primary events that contribute to the arterial lesions. Subsequently formation of foam cells and fatty streaks, and necrotic extracellular lipid ensue in these lesions. In later stages of inflammatory and pathological processes plaque formation occurs accompanied by the roles played by blood cells, endothelial cells and smooth muscle cells [37]. In 1993, Ross postulated that inflammatory response to the impaired intima and endothelium of arteries due to, e.g., mechanical injury, toxins, and oxygen radicals is the initiating event leading to endothelial dysfunction. Ross refers to atherosclerosis as a process in which endothelial inflammation is followed by the formation of fibrofatty and fibrous lesions [36]. Libby et al. has reviewed several clinical publications corresponding to atherosclerosis and suggest that, based on compelling evidences, inflammatory events and alterations of cellular and molecular mechanics are the underlying causes of atherosclerosis. Immune system response plays a key role in all stages of atherosclerosis. Scientific evidences favour the crucial role of inflammatory pathways in all stages of atherosclerosis as well as in arterial thrombosis [29]. All these authors, however, claimed the intimal layer of the artery to represent the anatomic structure first affected by the disease (“inside-ou” theory).

Atherosclerosis is a multifocal, smoldering, chronic immunoinflammatory disease of medium-sized and large arteries fuelled by lipids [17, 19, 29]. Atherosclerosis is by far the most frequent underlying cause of coronary artery disease, carotid artery disease, and peripheral arterial disease. The most devastating consequences of atherosclerosis, such as heart attack and stroke, are caused by superimposed thrombosis.

The genesis of the atherosclerosis is majorly influenced by endothelial cells, leukocytes, and intimal smooth muscle cells. Higher plasma cholesterol level in arteries is solely sufficient to promote atherosclerosis [17]. Experimental studies in mice suggest gene inactivation of immune cells’ receptor and immune cell (macrophage) colony-developing factor has significant influence on development of atherosclerosis [17]. Extravasation of plasma molecules and lipoprotein particles, depending on their size and concentration, through the leaky and impaired endothelium into the sub-endothelial space. In this space the atherogenic lipoproteins get retained, undergo changes (e.g., oxidation) and become cytotoxic, proinflammatory, chemotaxic, and proatherogenic. On set of atherosclerosis, the endothelial cells, macrophages and few T cells participate in developing the asymptotic foam cell-lesions and promote disease progression [11]. Inflammatory events play a vital role in atherogenesis. Mayerl et al. [30] have investigated atherosclerotic specimens from autopsies performed by Rokitansky up to 178 years ago using modern sophisticated immunohistochemical and immunofluorescence techniques. Their study revealed the presence of various cellular intralesional components—essentially CD3+ cells in early lesions as well as extracellular matrix proteins supporting Virchow’s view that primarily inflammatory arterial changes initiate atherogenesis. However, the crucial first pathogenetic events of the disease remained unclear. Classical concepts of atherogenesis did not attribute a major relevance to inflammatory immunologic processes as possible pathogenetic factors. The study based on their historical materials, suggests that inflammatory immunologic processes incite atherogenesis. However, the very first pathogenetic event remains to be elucidated [17].

In 1910, the German chemist, Windaus showed that atherosclerotic plaques consist of calcified connective tissue and cholesterol [42]. At the beginning of atherosclerotic changes in the vessel wall [34, 35], a stress protein—heat shock protein 60 (HSP 60) as the potential (auto) antigen incites an immune response. Classical atherosclerosis risk factors, such as high blood pressure, smoking, diabetes, biochemically modified LDL, etc., result in the expression of HSP60 by endothelial cells (EC), especially at sites that are subjected to major (turbulent) haemodynamic stress and known to be predilection sites for the later development of atherosclerotic lesions [35]. Diffuse Intimal Thickening (DIT) is intimal fibro-cellular thickening localized to the non-branching long arterial segments that spreads out circumferentially and longitudinally. DIT consists of two layers—the inner layer known as the proteoglycan layer because it contains ECM with abundant proteoglycans including SMCs while the outer layer is known as the musculoelastic layer because of the abundance of SMCs and elastic fibers with smaller amount of proteoglycans. Nakashima et al. examined coronary arteries of autopsy subjects with light microscopy and suggest that eccentric extracellular deposition of apolipoprotein B in the outer layer of DIT is the earliest stage of atherosclerosis (Type I lesion). Advanced stages of atherosclerosis is governed by macrophages infiltration in the deeper layer of the intima leading to formation of foam cells and eventually Type II lesions [34].

Kathryn and Tabas [33] suggest that accumulation of apolipoprotein B-lipoporotein (produced by liver and intestinal cells) in sub-endothelium space leads to recruitment of immune cells—monocytes, which differentiate into macrophages and dendritic cells. Williams and Tabas [41], based on their functional and morphological studies, claim that this accumulation is the primary initiation of atherosclerosis. Due to the deposition of cells, lipid and matrix, these macrophages incite the maladaptive, nonresolving inflammatory events causing the sub-endothelial layer to expand. As atherosclerotic lesions advances infiltration of smooth muscle and T cells into the intima takes place followed by amplification of apoB-lipoprotein retention. In succession lesions progress and form lipid filled necrotic core. Further progression of lesions makes them unstable and making plaques vulnerable to rupture leading to thrombotic vascular disease, myocardial infarction, stroke, and sudden cardiac death [33].

A plethora of studies on atherosclerosis suggests and supports the response to injury and inflammation theories involving endothelial dysfunction as incitement to the progression of atherosclerosis. The second author, based on his observations made in hundreds of cardiovascular surgeries, proposes that the disrupted or occluded vasa vasorum (VV—a network of small blood vessels that supply blood to the large blood vessel walls) would be the early underlying pathophysiological mechanism that triggers the inflammation and propagate from adventitia to intima [21]. In large and medium sized arteries VV are need to supply nutrition to vessel walls. Ischemic events in VV due to constriction precipitate fatty streaks in the underlying arterial segments [23]. This would be the prime initiation of atherosclerosis. Haverich, from his cardiac surgeries, observed no presence of adventitial VV in atherosclerosis-free arteries. Areas predicted as non-atherosclerotic in large and medium arteries possess hardly any VV implicating no probability of wall ischemic events. On the basis of this observation—lack of VV in areas spared from atherosclerosis, Haverich developed the unified theory—Atherosclerosis represents rather a micro vascular disease initiated by VV occlusion within the outer layer of the blood vessel (adventitia) that gets translated into arterial functional impairment (“outside-in” theory) [21]. The mathematical model developed by the first and third author is essentially based on the hypothesis proposed by the second author.

Unlike clinical investigations, the mathematical modeling and numerical simulation of the atherosclerosis, as a multi-physics problem, has rarely been undertaken. Khatib et al. developed 1D and 2D models to simulate the initiation of atherosclerosis triggered by inflammation. Inflammation induced by the diffusion (concentrations) of oxidized low density lipoproteins in the intima can lead to chronic inflammatory reaction (traveling wave propagation). High ox-LDL concentrations correspond to unstable systems [27]. Hidalgo and Tello have developed a 1D mathematical model of nonlinear diffusion for initial stage of atherosclerosis development. As the arterial wall is a porous medium, the nonlinear diffusion is added to the existing 1D model with linear diffusion, implying that the current model is an extension to model in [24]. Lie and Tand have developed a 3D mathematical model to simulate the plaque initiation and study the geometrical adaptation of atherosclerotic plaques. They proposed a linear plaque growth function that relates coronary artery diameter change and wall shear stress. This plaque growth function in combination with 3D Navier–Stockes (NS) equations have been solved numerically. Numerical studies emphasized on effects of WSS, blood viscosity and the inlet flow rate on the growth of atherosclerotic plaques. They observed plaque growth at decreasing rate as the atherosclerosis progresses. Results also indicated that a significant influence of the haemodynamic characteristics such as blood viscosity and the flow rate on the plaque growth [4].

A 3D computational model has been developed for plaque formation and progression for human carotid artery by Filipovic et al. [12]. In this model plaque growth is driven by LDL penetration in intima followed by inflammatory processes (recruitment of monocytes and cytokines). In this reference, the model utilizes various PDEs for mimicking various processes involved in atherosclerosis. This multi-scale model simulates blood flow with continuity and NS-equations and coupled with mass transfer into the arterial wall by a convection–diffusion equation. Kedem–Katchalsky equations are used to couple LDL transport in lumen and through vessel tissue. Furthermore, three additional reaction–diffusion PDEs have been incorporated for modeling inflammatory processes triggering the plaque growth. Functions of plaque growth volume are linked with distribution of shear stress and effective wall stress. The numerical observations on plaque localization correspond to low shear stress regions. The location and characteristics of plaque progression comply with in vivo observations. In a similar way, Cilla et al. [8] presents a multi-scale approach to model plaque formation and progression in coronary arteries. Cilla et al. consider WSS as the influencing contributor to the atherosclerosis and correlates therefore WSS to atherosclerotic plaques. Their mathematical model considers LDL, ox-LDL, monocytes, macrophages, foam cells, smooth muscle cells, cytokines and collagen as the contributors in early atherosclerosis development. PDEs incorporated in the model for fluid flow, mass balance and coupling are continuity and NS-equations, Darcy’s law, convection–diffusion–reaction equation and Kedem–Katchalsky equations. Numerical results performed on an axisymmetric geometrical coronary artery model show that this mathematical model can qualitatively predict the atherosclerotic lesion development in tunica intima. LDL concentration at the low WSS observation complies with clinical hypothesis.

Mirzaei and Fok [32] have developed a 2D mathematical model to study the behaviour of vessel wall in pre and post atherosclerosis. Morphoelasticity with anisotropic strain function is utilized to describe growth (a function of Platelet Derived Growth factor) in the mathematical model. Numerical simulations consider vessel wall as hyperelastic material by incorporating all three layers of arteries each assigned with its respective material properties. Resulting mechanical deformation and stress fields were studied in the intimal thickened regions due to underlying development of necrosis. Arterial thickening (morphological changes) from deformation behaviour were observed to comply with images acquired from ultrasounds scans. A 2D-mathematical model for simulating the initial pathophysiological processes of atherosclerosis was developed by Silva et al. [38]. This model represents a chronic disease that is strongly influenced by inflammatory events. The underlying mechanics such as dynamics of low-density-lipoproteins (LDL) and oxidized LDL, transport of monocytes from blood flow into the intima, and formation of foam cells due to macrophages ingestion of oxidized LDL have been incorporated. To consider the series of events from LDL accumulation (from blood flow) to foam cells formation in early stages of atherosclerosis as described in Fig. 2, the model uses and couples various PDEs (Navier–Stokes equations, Biot equations, convection/chemotaxisreaction–diffusion equations) accounting for specific processes involved. One of the essential element of this model is quantifying the endothelial permeability to LDL and to the monocytes as a function of wall shear stress (WSS), cytokines and LDL on the endothelial surface.

In this paper a coupled multi field continuum-based mathematical model is developed based on the newly presented hypothesis in [21]. As outcome of the model, for the first time, the mechanical deformation of the artery wall is initiated from the adventitial layer of the wall due to the disruption of nutrition flow in VV. In fact, the inflammatory response starts at the middle layers of the artery and propagates towards the innermost layer in an “outside-in” fashion. The formation a bulge as a result of inflammation give rise to the atherosclerosis and makes the lumen stenosed. The proposed model is novel in the sense that a phase-field approach has been employed to capture the tissue inflammation and its propagation. Additionally, the nutrient transport is also modeled in the presence of vasa vasorum network that serves as an auxiliary “perfusion” mechanism besides the classical “diffusion”. The large deformation of the artery is handled in a finite strain framework along with an anisotropic constitutive behaviour stemming from the collagen fibers in the structure of the artery.

## Mathematical Modeling of Atherosclerosis

**Fig. 1:**
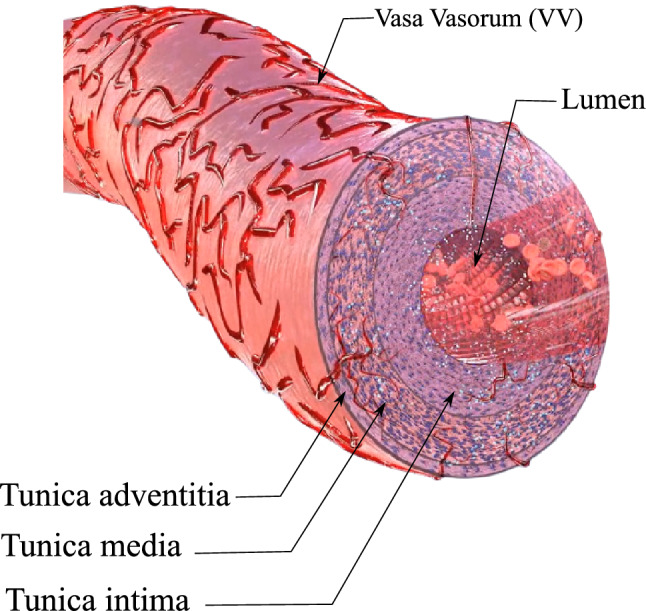
The structure of a typical elastic artery with vasa vasorum microvessels

From a biological point of view, the wall artery consists of 3 layers, namely intima, media and adventitia each of which is reinforced with circumferential collagen fibers. It is known that these layers differ in mechanical properties as well as the orientation of the collagen fibers, see Fig. 1.

The evolution of atherosclerosis is described using a coupled multi-field approach. The displacement field $\varvec{u}$ captures the mechanical deformation. The availability of the nutrient is represented by scalar variable *c* dedicated to the concentration of the nutrient. Finally the tissue inflammation (overgrowth) is denoted by $\phi$ that is treated as a phase-field variable. While the mechanical part is governed by the well-known momentum conservation law, the nutrient transportation obeys a classical diffusion–reaction equation. Nevertheless, the phase-field variable is treated in a so-called Allen–Cahn type phase field modeling. In order to establish a physically meaningful coupling between these fields, one needs to know the underlying “Assumptions” as well as the “Hypotheses” which are listed below:

**Fig. 2:**
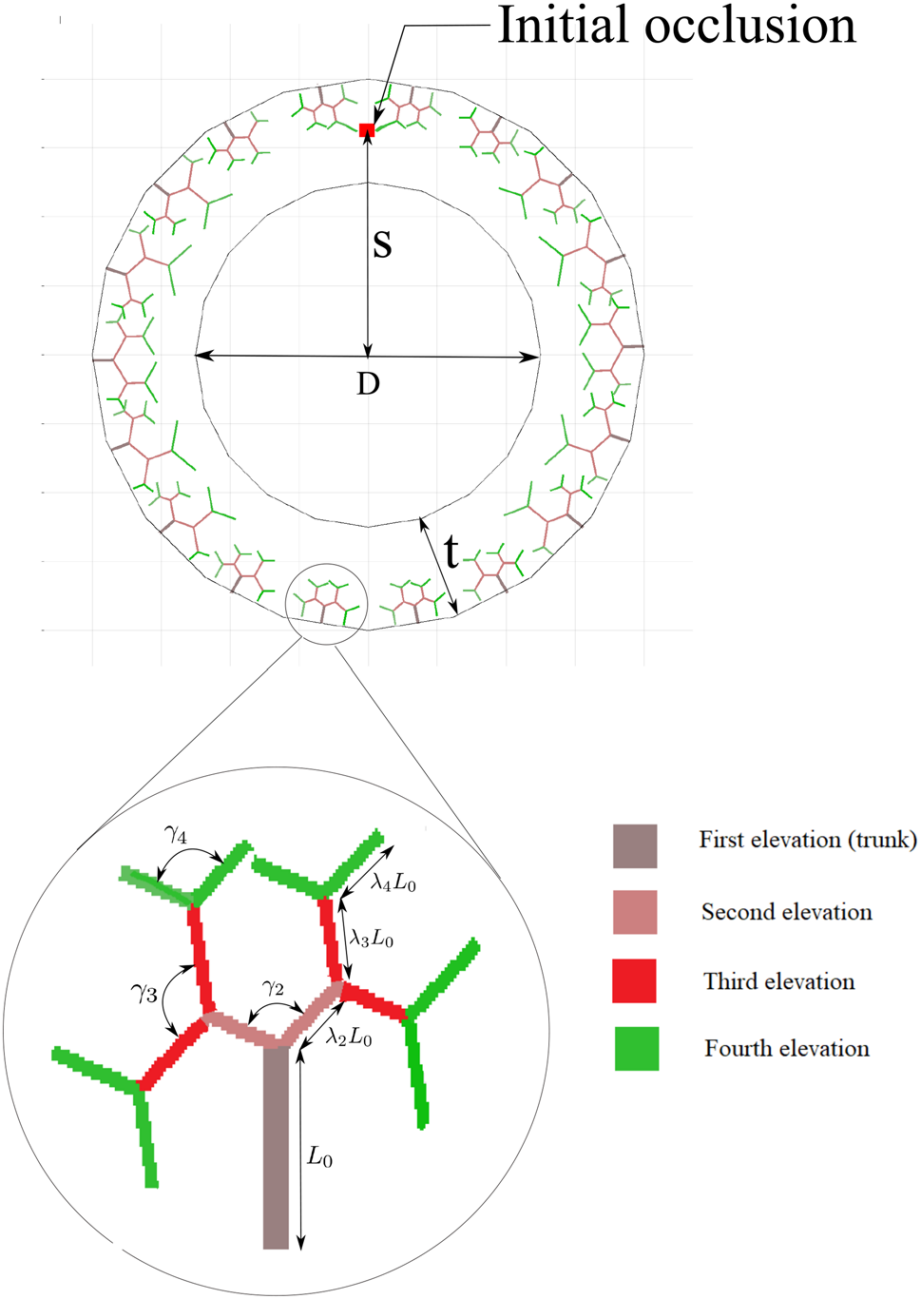
Geometrical representation of vasa vasorum using tree fractal concept and also initial occlusion

### **Assumptions**

1. For the sake of simplicity and avoidance of dealing with the discontinuities across the multi-layer structure, we do not make a distinction between the layers by assuming the same material properties and collagen orientations for all layers.
2. The artery issues close to the innermost layer (intima) are fed through the diffusion mechanism from the blood flowing in the lumen. Nevertheless, due to the thick wall of the artery the diffusion mechanism is not sufficient for the nourishment of the farther tissues with respect to the lumen. Hence, the outer layers of the artery are supplied with the nutrient via a network of capillaries called “vasa vasorum (VV)”, see Fig. 1. This network is created stochastically using tree-like fractals that starts at the exterior of the wall and penetrates up to the middle of the artery wall. A 2D illustration of this structure is shown in Fig. 2. The parameters that quantitatively control the geometrical shape of VVs are shown in this figure. An open-source MATLAB code [10] is utilized to create the structure of VVs.
3. The blood flow is not modeled explicitly, neither in the lumen nor in the vasa vasorums. Instead, it is replaced with the boundary conditions that are physically related to the blood flow. In particular, the value of the maximum nutrient is prescribed on the surfaces that are in direct contact with the blood. Furthermore, the systolic mean pressure of the blood in the lumen is applied on the innermost surface of the wall.
4. The consumption rate of the cells is assumed to be uniform in the entire domain (artery wall). This simplifies the reaction term of the nutrient transport equation leading to a minimum number of model parameters. Similarly, the diffusivity of the nutrient across the artery wall is taken to be constant prior to emergence of the inflammatory response. However, upon developing the inflammation we assume that the diffusivity decreases due to a histologically denser tissue resulting from the inflammation.
5. The mechanical deformation of the artery wall is formulated in a finite strain framework that accounts for the large deformation arising from the overgrowth phenomenon. For the mathematical framework of the growth, the classical and well-known multiplicative split of the deformation gradient is adopted.

The mathematical model proposed here for the atherosclerosis is based on three simple hypotheses:

### **Hypothesis 1**

**Fig. 3:**
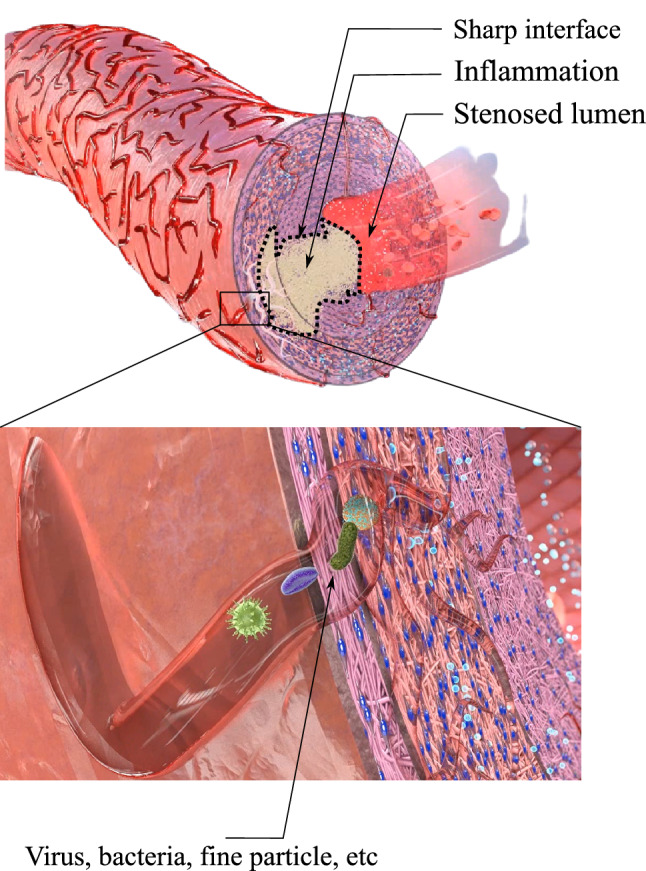
Inflammation as a response to the vasa vasorum occlusion due to viruses, bacteria and fine particle

The “inflammation (overgrowth)” is initiated and evolves in response to “nutrient scarcity”. From a biological point of view, as newly proposed in [21], the disruption of the nutrient flow within the outer layer of the wall tissue triggers the lesion evolution. As mentioned before, the occlusion of the vasa vasorum due to viruses, bacteria and very fine dust may lead to such situation, see Fig. 3. From a mathematical point of view, we introduce a threshold for the nutrient below which the inflammation starts. This postulate couples the nutrient transport equation to the phase field equation governing the inflammation.

### **Hypothesis 2**

The lesion develops in the direction of maximum nutrient change. Mathematically speaking, the boundaries of the inflammation, as a finite region, is advected in the direction of nutrient gradient. Keep in mind that the cell inflammation is captured using a phase field variable called $\phi$. If the cell is inflammatory it means $\phi =1$ and if the tissue is healthy it is represented by $\phi =0$. The sharp interface between these two regions can be perceived as the boundary of the lesion, see Fig. 3.

### **Hypothesis 3**

The overgrowth which is the mechanical reflection of the inflammation is proportional to the inflammation ($\phi$). The coupling between the mechanical deformation and the phase-field variable is established based on this hypothesis.

Now one can construct the governing equations based on the assumptions and the hypotheses listed above.

### Mechanical Equilibrium Equation

Since the atherosclerosis is a very slow process, the quasi static balance equation of the linear momentum in the absence of the body force is employed according to

1 $$\begin{aligned} \nabla \cdot {\varvec{\sigma }}=0, \end{aligned}$$

where ${\varvec{\sigma }}$ is the Cauchy stress and $\nabla \cdot$ refers to the divergence operator with respect to the spatial coordinate. We need to emphasize that the so-called “non-compliant” terms pertaining to the growth process are neglected in the balance equation of linear momentum due to the assumption of “slow growth” [18]. This is why the mechanical balance equation of the artery as a “viable system” is exactly similar to that of an “non-viable system”. In order to compute the stress tensor, the existence of a free energy function ($\Psi$) is postulated. One can write

2 $$\begin{aligned} {\varvec{\sigma }}=\frac{1}{J_e}\frac{\partial \Psi }{\partial {\varvec{F}}_{e}} {\varvec{F}}^T_e, \end{aligned}$$

in which ${\varvec{F}}_e$ designates the elastic part of the mechanical deformation with $J_e$ being its determinant ($J_e={\text {Det}}({\varvec{F}}_e)$). As mentioned in Assumption 4, the gradient deformation tensor ${\varvec{F}}$ is decomposed multiplicatively using

3 $$\begin{aligned} {\varvec{F}}= {\varvec{F}}_e {\varvec{F}}_g, \end{aligned}$$

in which ${\varvec{F}}_g$ is going to capture the overgrowth as a result of inflammation. Deformation gradient ${\varvec{F}}$ can be computed using the spatial gradient of the displacement field ${\varvec{u}}$, as a primal variable, according to

4 $$\begin{aligned} {\varvec{F}}=({\varvec{I}}-\nabla {\varvec{u}})^{-1}, \end{aligned}$$

where $\nabla$ signifies the spatial gradient operator. It will be utilized frequently.

For the free energy $\Psi$ a so-called poly convex function needs to be utilized in order to ensure the existence of a stable solution for the hyperelasic boundary value problem in hand, see [20]. Here a Holzapfel–Gasser–Ogden (HGO) type anisotropic hyperelastic free energy function [15] is utilized as follows

5 $$\begin{aligned} \Psi&=\underbrace{\frac{\mu }{2}(I_{1e}-3)}_{{\text {Matrix}}\,{\text{energy}}}+\underbrace{\frac{\eta }{\beta }e^{\beta [\rho <I_{4e}-1>^2+(1-\rho )(I_{1e}-3)^2]}}_ {{\text {Collagen}}\,{\text{fiber}}\,{\text{energy}}} \nonumber \\&\quad +\underbrace{\frac{\nu }{\mu (1-2\nu )}(J_e-1)^2-\mu {\text {Log}}J_e}_{{\text {Volumetric}}\,{\text{energy}}\,{\text{contribution}} }, \end{aligned}$$

with $I_{1e}$, and $I_{4e}$ being the invariants of the isochoric right Cauchy–Green tensor defined as ${\varvec{C}}_e=J^{-\frac{2}{3}}_e{\varvec{F}}_e^T {\varvec{F}}_e$. The parameters $\mu$, $\nu$, $\eta$, $\beta$ and $\rho$ signify the material parameters. One should note that this particular form of free energy function accounts for the well-known exponential stiffening response of the wall once its reinforcing wavy fibers reach their straitened length. Moreover, these fibers are active only when they are under tensile load. Hence, the so-called Macaulay’s parenthesis $<\bullet>$ appearing in Eq. (5) is defined as

6 $$\begin{aligned}< \bullet > = \left\{ \begin{array}{ll} \bullet &{}\quad {\text {If }}\, \bullet \ge 0\\ 0 &{}\quad {\text {If }}\, \bullet < 0 \end{array}\right. \end{aligned}$$

Finally, one can compute the invariants of ${\varvec{C}}_e$ using

7a $$\begin{aligned} I_{1e}&={\text {tr}}({\varvec{C}}_e) \end{aligned}$$

7b $$\begin{aligned} I_{4e}&={\text {tr}}({\varvec{n}} \cdot {\varvec{C}}_e \cdot {\varvec{n}}) \end{aligned}$$

in which the vector ${\varvec{n}}$ refers the direction of the collagen fiber at the point of interest. It is assumed that collagen fibers helically wound along the arterial axis. Vector ${\varvec{n}}$ is nothing else than the unit vector that is tangent to the helical curve. It is obvious that a generic vector ${\varvec{n}}$ in 3D space that vector can be expressed in terms of the azimuth angle ($\theta _{az}$) and elevation angle ($\theta _{el}$). Thus, one can write

8 $$\begin{aligned} {\varvec{n}} =[-{\text {Cos}}(\theta _{el}){\text {Sin}}(\theta _{az}),\ {\text {Cos}}(\theta _{el}){\text {Cos}}(\theta _{az}),\ {\text {Sin}}(\theta _{el})]. \end{aligned}$$

While $\theta _{az}$ changes in the interval [0, 2$\pi$] depending on the circumferential location of a point, the elevation angel ($\theta _{el}$) is assumed to be constant. It is actually a material parameter listed in Table 2.

One may refer to [13, 25] for more details regarding this particular form of free energy function and the underlying assumptions.

### Nutrient Transport Equation

It is assumed that the transport of the nutrient in the artery wall obeys the classical diffusion–reaction equation as follows

9 $$\begin{aligned} \nabla \cdot (D\nabla c)-R_c=0, \end{aligned}$$

where *c* represents the nutrient concentration and $R_c$ refers to the rate of the nutrient consumption in the cells. It is, indeed, the sink term for this equation. As stated in Assumption 4, $R_c$ is held constant and uniform (all the cells consume the nutrient equally). Besides, *D* denotes the diffusivity coefficient. It is assumed that the inflammation leads to a reduction in the nutrient diffusivity. The simplest transition model for the diffusivity coefficient can be established using a liner equation depending on the inflammation state $\phi$.

10 $$\begin{aligned} D=\phi D_{min} + (1-\phi )D_{max}, \end{aligned}$$

where $D_{max}$ and $D_{min}$ correspond to the diffusivity in the healthy and inflammatory tissue, respectively.

#### *Remark*

 The time scale of the diffusion process is substantially smaller than that of the inflammation process. In fact, the diffusion process is so fast that it reaches its steady state quickly during the extremely slow inflammation. This is why the time dependent term is eliminated from the nutrient transport equation.

### Inflammation Phase-Field Equation

Whether a particular region of the tissue has undergone inflammation process or not is captured using a phase-field variable called $\phi$. From a mathematical point of view, $\phi$ is, indeed, a binary marker showing the state of inflammatory status at the point of interest. Hence it is bounded in the interval [0, 1]. While $\phi =0$ means no inflammation, $\phi =1$ corresponds to inflammation occurrence. The sharp interface between 0 and 1 characterize the boundary of inflammatory cells.

The phase-field method has proven to be one of the most elegant ways in dealing with multiphasic problems (in particular, biphasic systems) in which the distinction between the phases matters and the objective is to capture the interface. In such problems, a secondary phase is embedded in in a primary phase and the boundary between the phases dynamically changes as a result of phases evolution. Since its advent in 1961 for binary alloys decomposition [7], phase field has successfully applied to image inpainting [3], crack propagation within a solid [31], multiphase flow [2], topology optimization [5] and solidification of a molten material [9]. Nevertheless, this method is relatively new to the computational biomechanics community. Although some researchers have employed phase-field in modeling the tumor growth [43], to the best of the authors knowledge, this work is the first attempt in which the phase field approach is utilized to model the evolution of atherosclerosis.

The central idea is that the interface between the inflammatory cells and the surrounding healthy tissue is to be captured by a phase field model of the type Allen–Cahn [1] as follows

11 $$\begin{aligned} \underbrace{\frac{\partial \phi }{\partial t}}_{\begin{array}{c} {\text {Phase}}\,{\text{evolution}} \\ {\text {in}}\,{\text{pseudo}}\, {\text{time}} \end{array}}=\underbrace{-Mf^{\prime}(\phi )}_{\begin{array}{c} {\text {Bulk}}\,{\text{contribution}} \\ \end{array}}+\underbrace{\epsilon ^2 \nabla ^2 \phi }_{\begin{array}{c} {\text {Sharp}}\,{\text{interface}} \\ {\text {contribution}} \end{array}}+\underbrace{S(\phi ,c)}_{\begin{array}{c} {\text {Driver}}\,{\text{(source)}} \\ {\text {of}}\,{\text{ phase-field}} \end{array}}, \end{aligned}$$

in which the two parameters $\epsilon$ and *M* are meant to control the width of the interface between the phases and the energy jump therein, respectively. $f^{\prime}(\phi )$ is a short notation for $\frac{\partial f(\phi )}{\partial \phi }$. The description of each term in the equation is self-explanatory and standard in any application of the phase-field modeling. The function $f(\phi$) is a so-called double-well potential and characterizes the barrier to be overcome in order to have a phase transformation (here, phase transformation means a healthy tissue becomes inflammatory). It is a common choice to take

12 $$\begin{aligned} f(\phi )=16M \phi ^2(1-\phi )^2, \end{aligned}$$

in which $M=f(\frac{1}{2})$ is the local maximum value of the function between the two wells at $\phi =1$ and $\phi =0$.

The corner stone of the phase-field modeling is how to define the source term $S(\phi ,c)$ which is, in fact, the driver of the phase field. It should be thermodynamically consistent or, at least, physically meaningful. For example, in the crack propagation application, there exist a well-established and thermodynamically consistent expression for this term using the elastic energy associated with the tensile stresses that make the crack grow. Here we use a “physically meaningful rationale” based on Hypothesis 2. The function $S(\phi ,c)$ can be written as

13 $$\begin{aligned} S(\phi ,c)=R_s H(c-c_{cri})\frac{\nabla \phi \cdot \nabla c}{|\nabla c|}, \end{aligned}$$

where $R_s$ is a parameter that controls the magnitude of the source term. The function $H(c-c_{cri})$ is the Heaviside step function with a jump at critical concentration $c_{cri}$. A general definition of $H(\bullet -\bullet _{cri})$ with a jump at $\bullet _{cri}$ is defined according to

14 $$\begin{aligned} H(\bullet -\bullet _{cri}) =\left\{ \begin{array}{ll} 1&\quad {\text {If }}\, \bullet \le \bullet _{cri} \\ 0 &\quad {\text {If }}\, \bullet > \bullet _{cri}. \end{array}\right. \end{aligned}$$

We shed more light on the structure of function $S(\phi ,c)$ due to the fact the novelty of this work relies on that. The role of function $H(c-c_{cri})$ is to activate the source term $S(c,\phi )$ only in case of the nutrient concentration drops below a critical value, namely $c_{cri}$. This function accommodates the first hypothesis in the construction of $S(\phi ,c)$. Furthermore, the term $\frac{\nabla c}{|\nabla c|}$ is a unit vector pointing to the direction of maximum change in the nutrient. Considering the second hypothesis, the dot product of this vector and $\nabla \phi$ is an advection-like term which tends to move the interface. Now, it is obvious that $R_s$ is nothing else than the magnitude of the advection velocity.

#### *Remark*

 The authors are aware of the torturous complexity arising naturally from the advection term in form of ${\varvec{v}}\cdot \nabla \phi$ with ${\varvec{v}}=K_s H(c-c_{cri})\frac{\nabla c}{|\nabla c|}$ being the advection velocity. An unsymmetric contribution to the stiffness matrix is one of the cumbersome consequences. Furthermore, if the divergence of ${\varvec{v}}$ is positive, it leads to the loss of coercivity in the corresponding billinear of this equation and ultimately to stability issues, see [26, 40]. If the advection term becomes dominant, the resulting instability becomes so tenacious that it necessitates employing particular numerical remedies. Since the inflammatory process is slow, we can use small values for $R_s$ and hence the numerical code does not fail even in the absence of stabilizing terms.

The closure of the mathematical modeling is to invoke the third hypothesis and link the mechanical part of the growth tensor ${\varvec{F}}_g$ to the inflammation state $\phi$. If ${\varvec{F}}_g$ is assumed to be isotropic, it can be characterized by a scalar $\alpha$ and the identity tensor $\varvec{I}$, as follows

15 $$\begin{aligned} \varvec{F}_g=(1+\alpha ) \varvec{I}. \end{aligned}$$

Here, the scalar $\alpha$ is introduced to capture the overgrowth. The initial value of $\alpha$ is set to be zero. The velocity gradient corresponding to the rate of the growth tensor can be computed using

16 $$\begin{aligned} {\varvec{L}}_g=\dot{\varvec{F}}_{g} \varvec{F}_{g}^{-1}=\frac{\dot{\alpha }}{1+\alpha }{\varvec{I}}, \end{aligned}$$

in which the time derivative is denoted by a dot overhead. The parameter $\alpha$ is treated as an internal variable. As an intuitive and simple choice, we postulate that the overgrowth magnitude is linearly proportional to the inflammation state according to

17 $$\begin{aligned} \frac{\dot{\alpha }}{1+\alpha }=k_g H(\alpha -\alpha _{cri}) \phi , \end{aligned}$$

in which $k_g$ is the proportionality coefficient that is, in fact, a model parameter. The Heaviside function $H(\alpha -\alpha _{cri})$ [defined in Eq. (14)] is introduced to prevent the variable $\alpha$ from growing unboundedly. In other words, $\alpha$ cease to increase further if it reaches the critical value $\alpha _{cri}$. One should note that among several other choices, this approach is only one of the simplest method to mathematically regulate a variable and keep it bounded. Without the imposition of limiting constraints on the growth function, it can literally approach infinity that is physically inadmissible.

## Numerical Implementation Using FEM

In order to implement the equations discussed in the previous section, we adopt a standard Galerkin FEM. Combining the week form of Eqs. (1), (10) and (11). One can construct a Lagrangian ${\mathcal {L}}$ as a function of primary variables ${\varvec{u}}$, *c* and $\phi$ whose stationary conditions retrieve those set of equations. It reads

18 $$\begin{aligned}&\delta {\mathcal {L}}({\varvec{u}},c,\phi )=\int _{\mathcal {B}} (\nabla \cdot {\varvec{\sigma }})\cdot \delta {\varvec{u}} \ dv+ K_c \int _{\mathcal {B}} [\nabla \cdot (D \nabla c ) \delta c+R_c \delta c] dv \nonumber \\&\quad +K_{\phi }\int _{\mathcal {B}} [(\epsilon ^2\nabla \cdot \nabla \phi ) \delta \phi +M f^{\prime}(\phi )\delta \phi -S(\phi ,c)\delta \phi +\dot{\phi }\ \delta \phi +K_p {\mathcal {P}}^{\prime}(\phi ) \delta \phi ]\ dv=0 \end{aligned}$$

where the parameters $K_{\phi }$ and $K_c$ are are numerical parameters introduced to obtain a good condition number of the final multi-field stiffness matrix. Their choice strongly affects the performance of the monolithic approach in solving the multi-field system. Furthermore, $K_p$ is the penalty parameter for the penalty function ${\mathcal {P}}(\phi )$ that enforce the boundedness of the phase-field variable $\phi$ in the interval [0, 1]. The penalty function ${\mathcal {P}}(\phi)$ whose derivative appears in Eq. (18) can be defined using Macaulay’s parenthesis defined in Eq. (6) and commonly used quadratic terms according to

19 $$\begin{aligned} {\mathcal {P}}(\phi )=<\phi -1>^2+<-\phi >^2 \end{aligned}$$

Upon applying the integration by parts to the integrals in Eq. (18), the boundary terms defined on the boundary $\partial {\mathcal {B}}$ emerge. Furthermore, a backward (implicit) Euler scheme is invoked in order to express the only temporal term ($\dot{\phi }$) in a finite difference manner. Altogether this leads to

20 $$\begin{aligned}&\int _{\mathcal {B}} {\varvec{\sigma }}: \nabla ^{sym} \delta {\varvec{u}} \ dv \nonumber \\&\quad +K_c \int _{\mathcal {B}} [D \nabla c \cdot \nabla \delta c\ +R_c \delta c] \ dv \nonumber \\&\quad +K_{\phi }\int _{\mathcal {B}}[\epsilon ^2\nabla \phi \cdot \nabla \delta \phi +M f^{\prime}(\phi )\delta {\phi }+\frac{\phi -\phi ^{n-1}}{\Delta t} \delta \phi \nonumber \\&\quad -S(\phi ,c)\delta \phi +K_p {\mathcal {P}}^{\prime}(\phi ) \delta \phi ]\ dv \nonumber \\&\quad -\int _{\partial {\mathcal {B}}} {\varvec{t}} \cdot \delta {\varvec{u}} \ ds -K_c \int _{\partial {\mathcal {B}}} D \nabla c\cdot {\varvec{n}} \ \delta c\ ds \nonumber \\&\quad -K_{\phi }\int _{\partial {\mathcal {B}}} \epsilon ^2\nabla \phi \cdot {\varvec{n}} \ \delta \phi \ ds=0 \end{aligned}$$

where ${\varvec{n}}$, that appears in the boundary integrals, is the normal vector of the boundary surface. It is a common and justifiable assumption that the boundary flux term for the phase-field variable is prescribed to zero. Hence, one can drop the term $K_{\phi }\int _{\partial {\mathcal {B}}} \epsilon ^2\nabla \phi \cdot {\varvec{n}} \ \delta {\varvec{\phi }}\ ds$. Moreover, ${\varvec{t}}={\varvec{\sigma }} \cdot {\varvec{n}}$ denotes the traction (mechanical flux) applied on the boundaries. Similarly, $D \nabla c. {\varvec{n}}$ refers to the nutrient flux at the boundary.

The implementation of the multi-field problem (mechanical deformation, nutrient concentration and the inflammation) in hand has been carried out using Ace-Gen, see [28] which is a powerful tool in automatic differentiation (hybrid symbolic/numeric differentiation). The generated FORTRAN code can be invoked by any FEM solver. Here we selected ANSYS due to its rich pre-processor and post-processor features. The topology of the element is a regular 3D brick element with linear Ansatz function and 8 nodes. Each node has five degrees of freedom. Three of them represent the displacement vector ${\varvec{u}}$ components. The remaining two are allocated to the nutrient concentration field *c* and the phase field variable $\phi$. Furthermore, at the Gauss points the variable $\alpha$ is treated as an internal variable. It is assumed that all internal and field variables are known at the previous time step. This is underlined using an *n* subscript for those variables. An implicit iterative procedure is utilized based on Newton–Raphson method. The solution of the global system yields the current values for the primary variables, namely ${\varvec{u}}$, *c* and $\phi$. The current values are denoted using $n+1$ superscript. The implemented algorithm is summarized in Table 1.

**Table 1:**
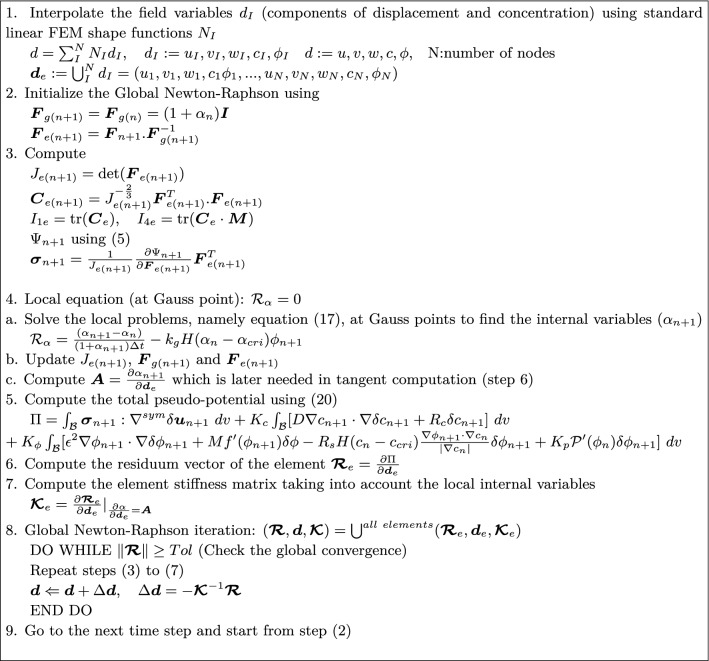
Implementation algorithm in the AceGen

## Numerical Examples

**Fig. 4:**
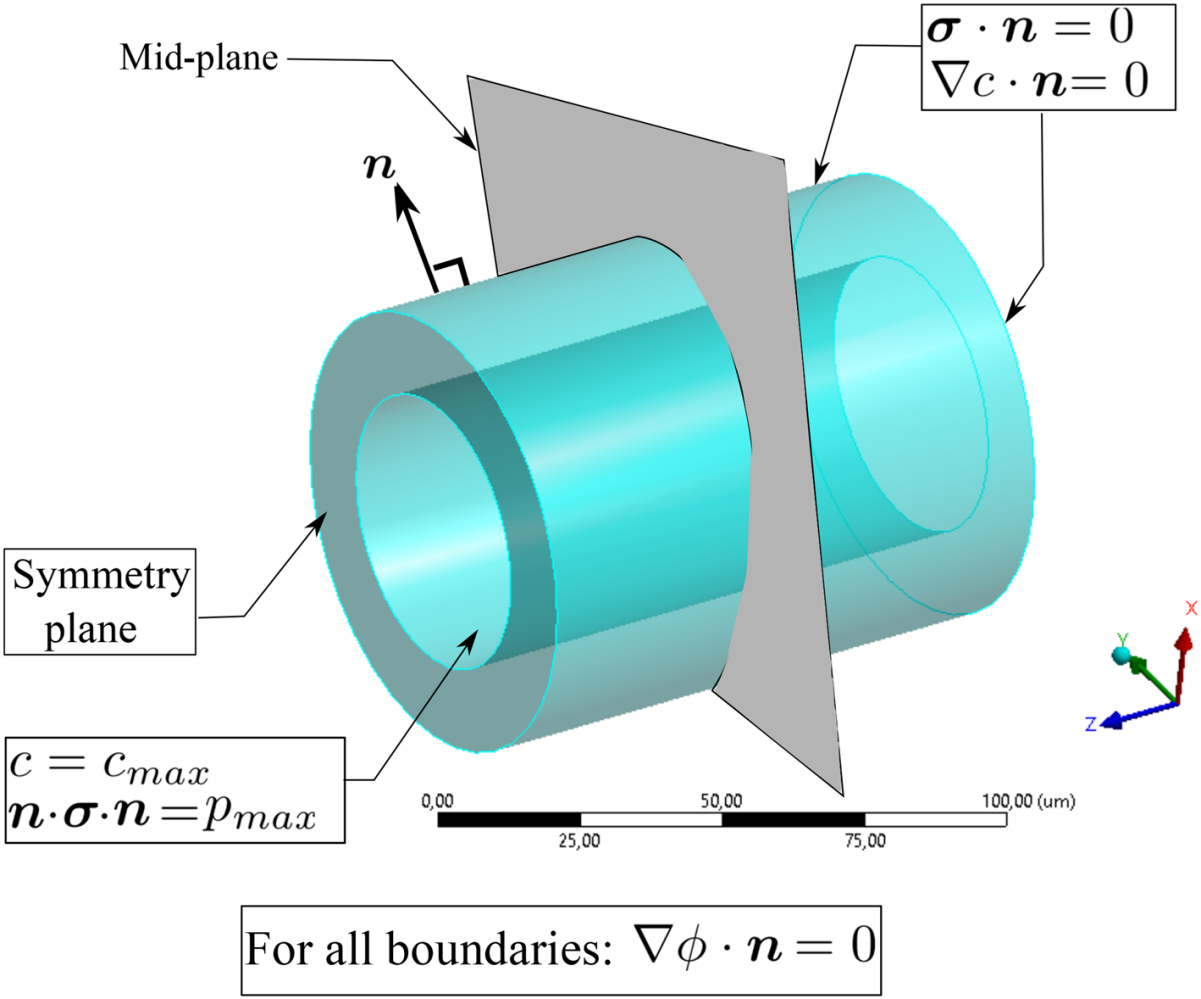
Boundary conditions an the artery for the multi-field problem

Two and three-dimensional examples are considered to show the features of the new framework for the prediction of the atherosclerosis development. The 2D examples concentrate on the verification of the model, while the 3D example demonstrate the entire evolution process. All geometrical data as well as numerical/material parameters are listed in Table 2. Moreover, the boundary conditions setting is illustrated in Fig. 4.

**Table 2:** Geometrical parameters of the test cases and material constants

Discreption	Parameter	Value	Unit
Inner diameter	D	50.0	$\upmu{\mathrm{m}}$
Wall thickness	t	15.0	$\upmu{\mathrm{m}}$
Artery length	L	100.0	$\upmu{\mathrm{m}}$
Initial occlusion location	S	30.0	$\upmu{\mathrm{m}}$
Fiber helix angle (elevation)	$\theta _{el}$	60	Degree
Overgrowth constant	$k_g$	0.25	${\text {Time}}^{-1}$
Max. concentration	$c_{max}$	1.0	$\upmu{\mathrm{g}}\,\upmu{\mathrm{m}}^{-3}$
Max. internal pressure	$p_{max}$	3.3	kPa
Cell consumption	$R_c$	$10^{-3}$	$\upmu{\mathrm{g}}\, \upmu{\mathrm{m}}^ {-3}\,{\text {Time}}^{-1}$
Max. diffusion coefficient	$D_{max}$	1.0	$\upmu{\mathrm{m}}^2\,{\text {Time}}^{-1}$
Min. diffusion coefficient	$D_{min}$	0.1	$\upmu{\mathrm{m}}^2\,{\text {Time}}^{-1}$
Critical nutrient concentration	$c_{cri}$	1.0	$\upmu{\mathrm{g}}\, \upmu{\mathrm{m}}^{-3}$
Critical growth	$\alpha _{cri}$	$10^{-3}$	–
Inflammation rate	$R_s$	0.1	$\upmu{\mathrm{m}}\,{\text {Time}}^{-1}$
Shear modulus	$\mu$	10	kPa
Poisson ratio	$\nu$	0.49	–
Free energy parameter	$\eta$	100	kPa
Free energy parameter	$\beta$	1	–
Free energy parameter	$\rho$	0.5	–
Phase-field parameter	$\epsilon$	1	–
Phase-field parameter	*M*	10	-
Phase-field penalty parameter	*Kp*	$10^{4}$	–
Numerical parameter	$K_{\phi }$	1	–
Numerical parameter	$K_c$	1	–
Mesh size		0.5	$\upmu{\mathrm{m}}$

### 2D Simulation of Atherosclerosis in an Artery

**Fig. 5:**
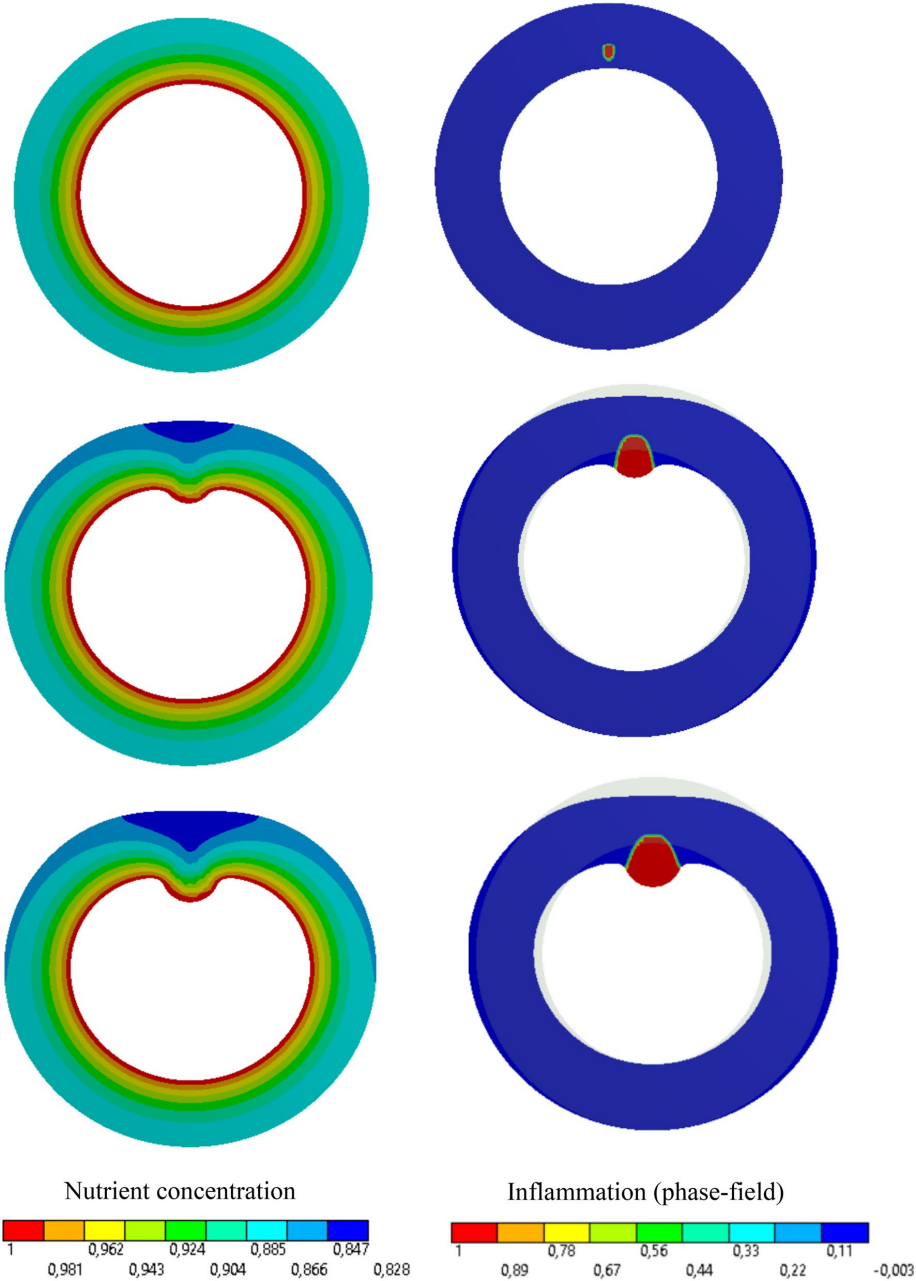
Variation of dimensionless nutrient and inflammation progression in the course of the time (without collagen fiber/without vasa vasorum)

#### Artery Without Collagen Fibers and Without Vasa Vasorum

In this example, a 2D representation of the artery (artery cross section at the mid-plane shown in Fig. 4) is modeled. This is the simplest case where both collagen fibers and vasa vasorum network are excluded from the analysis so that one can verify the numerical tool. The artery has neither collagen fibers nor the vasa vasorum. It is known that this scenario is the case for some so-called *muscular* arteries that are generally small-sized. Since these arteries are generally far from the heart and not subjected to relatively high pressure, they do not have any fiber. Moreover the nutrient diffusion from the lumen suffices due very thin wall thickness. From a modeling point of view, this simplification is translated in disregarding the fiber-associated term in the free energy function. In other words, the constitutive behaviour of the artery wall is isotropic. In the next test case, the impact of reinforcing collagen fibers is investigated in terms of wall deformation. As shown in the Fig. 4, the maximum concentration is prescribed on the inner surface of the artery. Furthermore, an initial nutrient perturbation (nutrient depletion) is artificially inserted at the location shown in this figure. This serves as the initiation of the phase-field parameter that is going to capture the inflammation. Figure 5 shows the snapshots of lesion development as well as the deformation of the artery wall. One can observe that the inflammation front advances dominantly in the radial direction where the nutrient concentration is maximal.

**Fig. 6:**
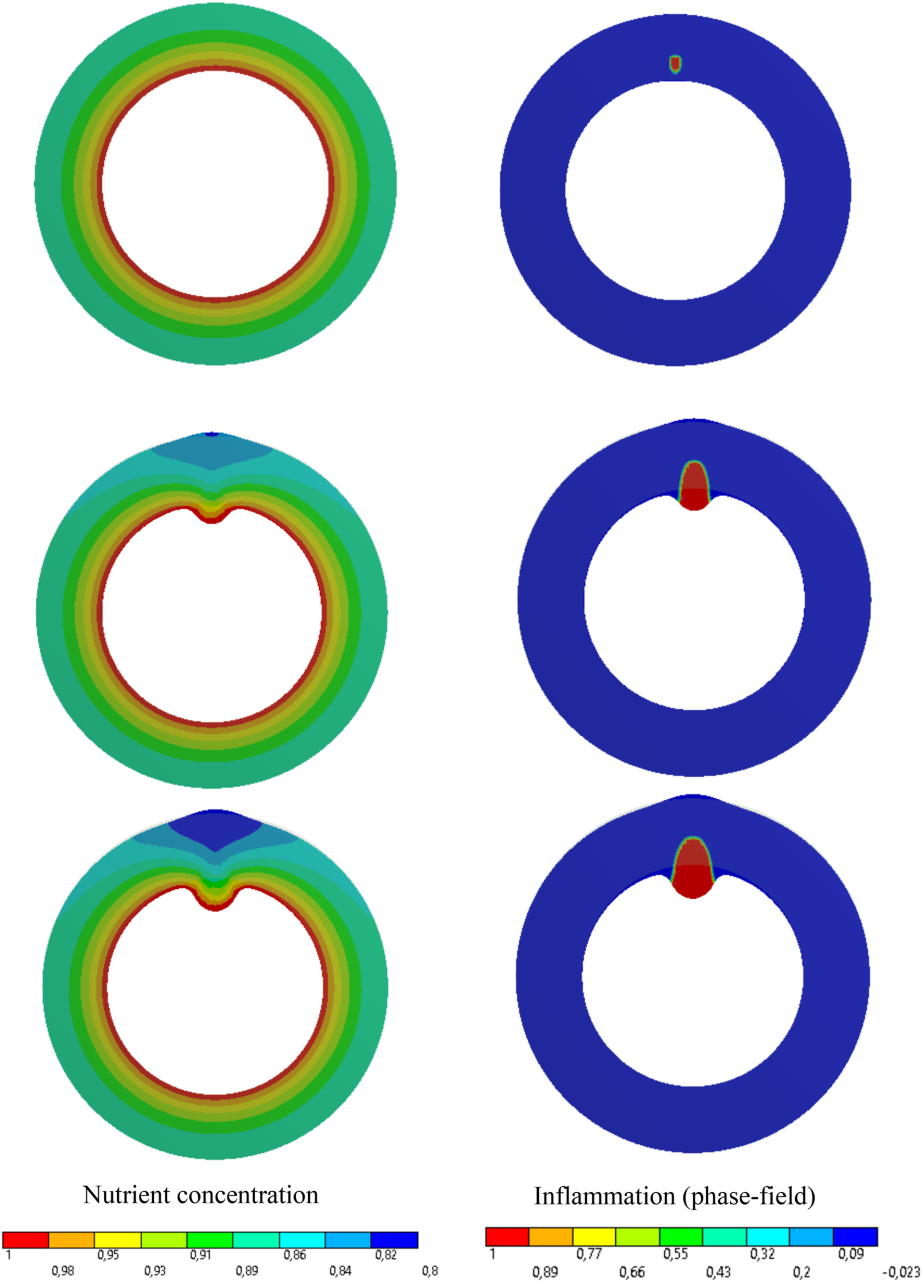
Variation of dimensionless nutrient and inflammation progression in the course of the time (with collagen fiber/without vasa vasorum)

#### Artery with Collagen Fibers and Without Vasa Vasorum

For this example, all material parameters are the same as the previous one except for those associated with the fiber contribution in the free energy. It is assumed that the fibers are placed circumstantially and in the plane of 2D cross section ($\theta _{el}=0$). The boundary and initial conditions remain intact. This way one can clearly see the impact of the reinforcing collagen fibers on the formation of the bulge due to the inflammation. Analogous to the previous test case, in Fig. 6 one can observe the inflammatory response of the artery wall as a result of lesion development. The interesting observation is that the collagen fibers reinforce the artery wall in the circumferential direction and hence the bulge tends to be guided in the radial direction both inward and outward.

**Fig. 7:**
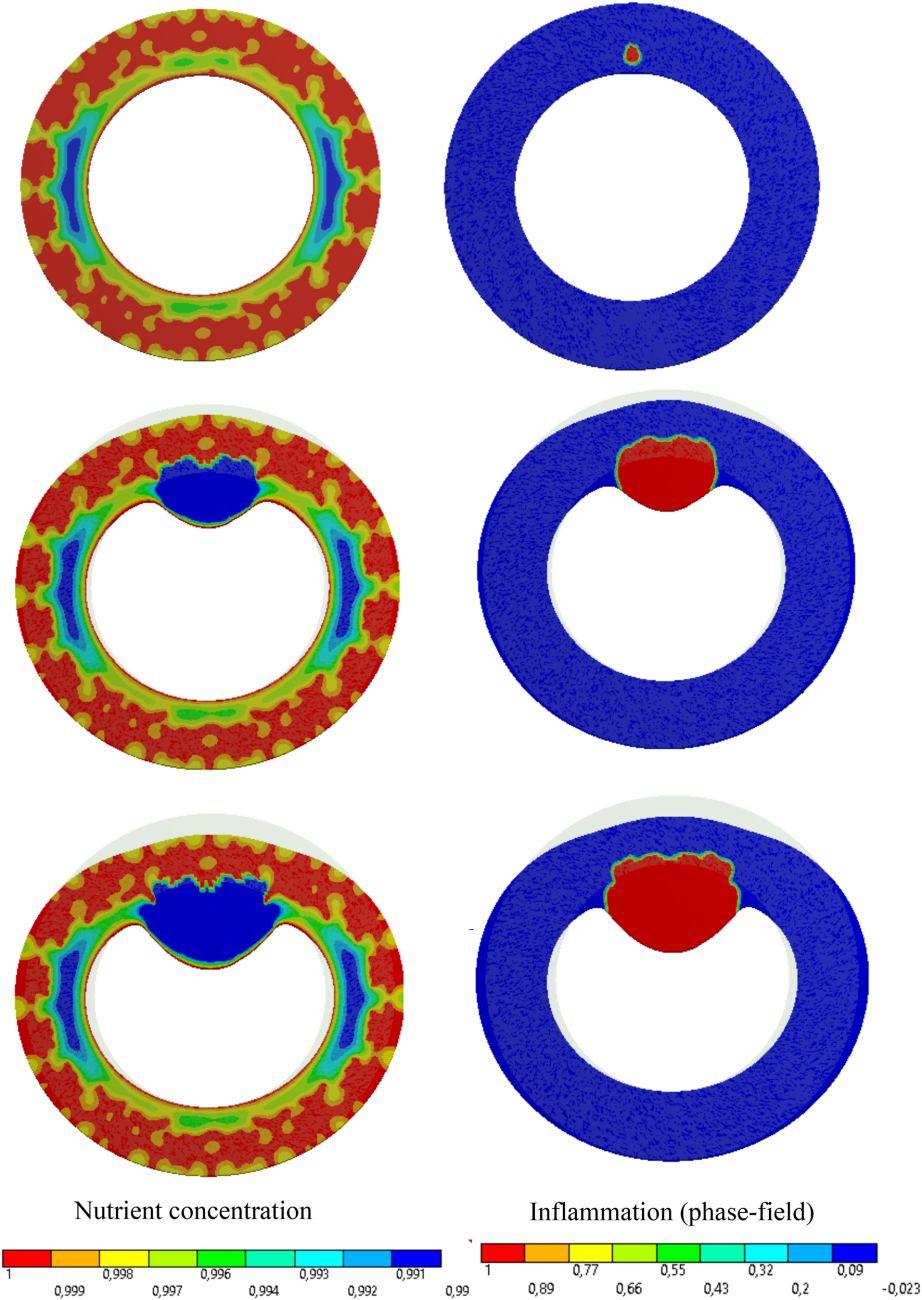
Variation of dimensionless nutrient and inflammation progression in the course of the time (with collagen fiber/with vasa vasorum)

**Table 3:** Geometrical parameters of vasa vasorum tree fractal

Discreption	Parameter	Value	Unit
Tree trunk	$L_0$	3	$\upmu{\mathrm{m}}$
Second branching parameter	$\lambda _2$	1.0	–
Third branching parameter	$\lambda _3$	1.0	–
Forth branching parameter	$\lambda _4$	1.0	–
Second branch angle 2D	$\gamma _2$	$\frac{2\pi }{3}$	–
Third branch angle 2D	$\gamma _3$	$\frac{2\pi }{3}$	–
Forth branch angle 2D	$\gamma _4$	$\frac{2\pi }{3}$	–
Azimuth angles for 3D branching	$\gamma _{az}$	$[0, \frac{2\pi }{3}, \frac{4\pi }{3}]$	–
Elevation angles for 3D branching	$\gamma _{el}$	$[\frac{\pi }{3}, \frac{\pi }{3}, \frac{\pi }{3}]$	–

#### Artery with Both Collagen Fibers and Vasa Vasorum

In the last 2D case, we have included both collagen fibers and vasa vasorum in the model. To accommodate the role of the vasa vasorum network, one needs to find all elements that are cut by tree fractals representing the vasa vasorum network. The tree fractal is generated using a MATLAB algorithm. The parameters that control the structure of the network are listed in the Table 3. In order to introduce some stochastic features to the formation of the network, some random values can be chosen according to the mean value and the standard deviation. Undoubtedly, one can have a more decent and realistic representation of the vasa vasorum by utilizing real microscopic images and reconstruction techniques. Here, the focus is on the “applicability” of the proposed model, rather than using the real clinical data. As the future extension of this work, one can certainly apply the real histological data of the artery.

The vasa vasorum network affects the nutrient field directly because they are, indeed, tiny vessels supplying the wall with the nutrient in addition to the diffusion mechanism. In practice, the maximum value of the nutrient concentration is prescribed on all elements lying on the tree fractal. In other words, the presence of vasa vasorum is equivalent to the modification of the boundary condition for the nutrient transport equation.

The interesting outcome of vasa vasorum model is that it substantially changes the nutrient distribution and consequently its gradient. Since the phase field parameter, that captures the inflammation, is derived and regulated by the nutrient gradient, one can notice a significant change in the appearance of the pathology. Figure 7 depicts the progression of the inflammation in the presence of vasa vasorums. All the parameters were kept as in the two previous examples. An irregular shape of the inflammation region is reproduced naturally due to the application of phase-field approach. Loosely speaking, the inflammation spreads spatially as if if would crawl in the ducts with the “lower nutrient level”. One can see that it turns around the regions nourished sufficiently by the vasa vasorums. One can find that the phase-field modeling reveals its beauty and amazing strength in capturing such phenomena. There is no need for interface tracking. The sharp interface between the binary phases (inflammation and the surrounding tissue) is self-produced. Dealing with evolving irregular binary phases of biphasic problems is highly challenging if one intends to track the interface explicitly and to enforce the compatibility condition across it.

**Fig. 8:**
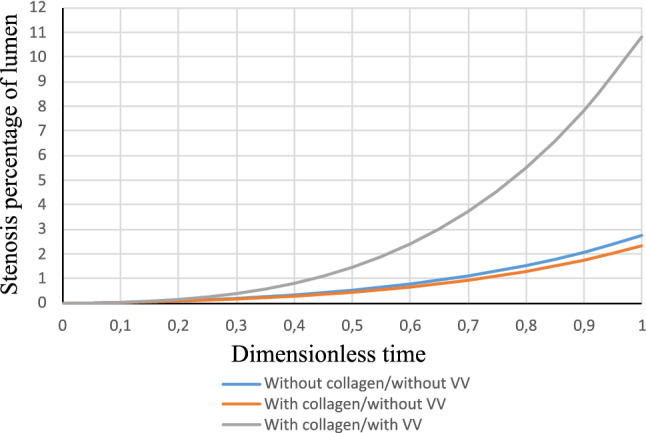
Percentage of reduction in the lumen cross section (stenosis degree) for different cases

In order to have a comparative and quantitative study between the three cases discussed above, we plot the percentage of the stenosis related to three cases in Fig. 8. It reflects to what extent the lumen cross section has reduced due to the emergence of atherosclerosis. One can extract two important results from this graph. First, the positive role of collagen fibers in making the artery more resilient to the lumen area reduction. The second interesting phenomenon is that the presence of vasa vasorum influences the wall response in favor of the stenosis. We found that the vasa vasorum affects the inflammation pattern by diverting the lesion spread into an “enlarged and dendritic” pattern from a purely “radial and compact” morphology. As a consequence, the bulge creation is not as in tense as the case without vasa vasorum. Furthermore, the abundance of nutrient as a result of vasa vasorum-assisted nourishment hinders the development of the inflammation. One should not forget that the inflammation starts and spreads as a result of nutrient scarcity.

### 3D Simulation of Atherosclerosis

**Fig. 9:**
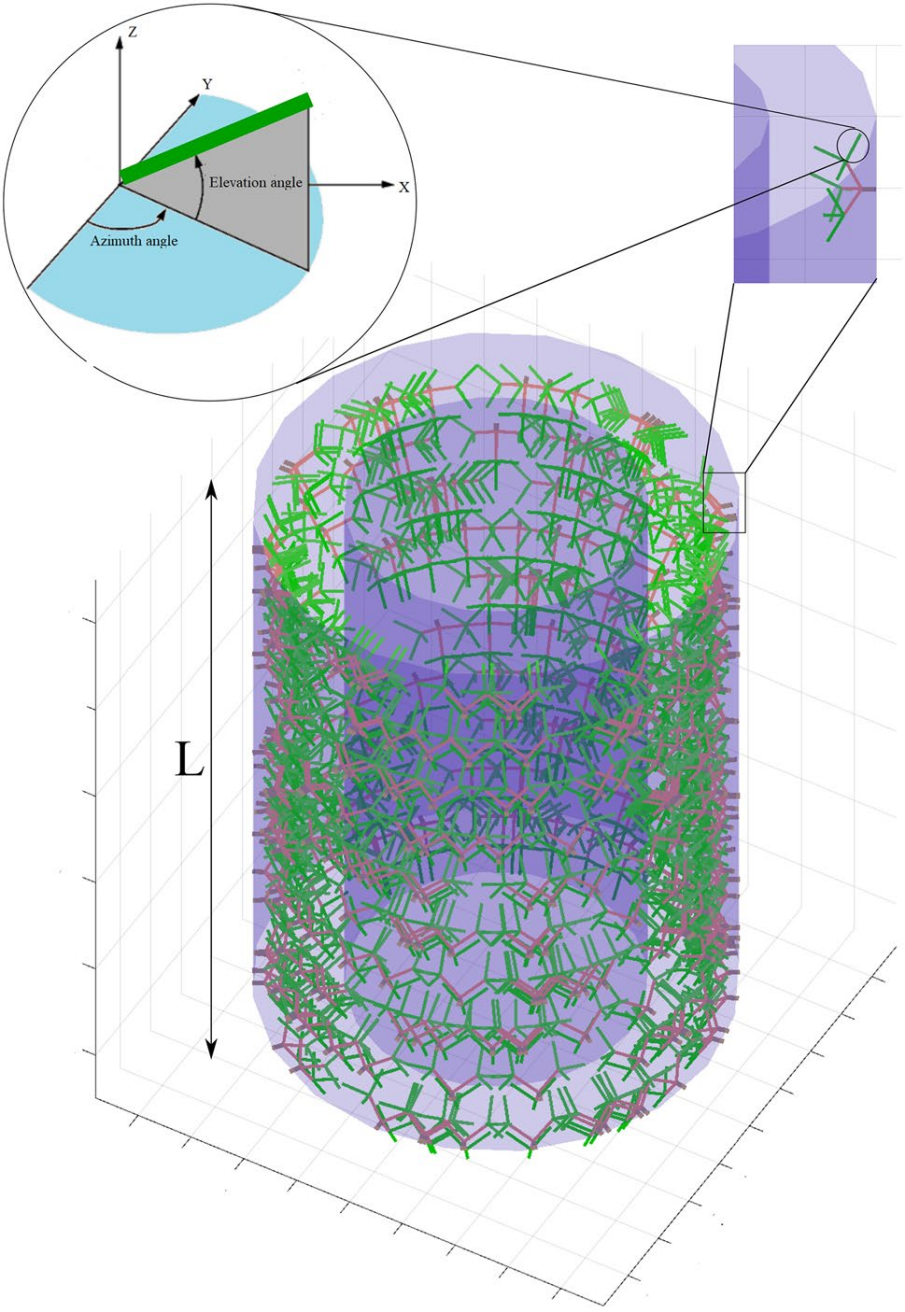
Geometry of the vasa vasorum in 3D

The mathematical modeling and the numerical implementation have been conducted in a 3D framework. Hence the final test case is dedicated to a full 3D model of the atherosclerosis. Figure 9 displays a randomly generated network of vasa vasorum in a 3D fashion. Establishment of proper boundary conditions plays an important role in this example. One should keep in mind that the artery model with a finite length here should be as close as possible to the physiological conditions under which the artery functions. Isolation of a cut partition of the artery from the original artery network should be replaced with “appropriate” boundary conditions, see Fig. 4. The reason is that it used to be a part of a longer artery embedded in the surrounding tissues. For example, if the artery is mistakenly and fully constrained in the axial direction, the overgrowth may lead to intense compressive loads and finally buckling of the artery that is not physical. Hence, the axial freedom of the artery needs to be insured to avoid such artifacts. The impact of the background elastic tissue, that serves as an elastic matrix for the artery, is neglected in this work.

In principal, it is known that the arteries are not necessarily stress-free even in load-free state. it manifests itself in the emergence of an open sector if the artery is radially cut in the laboratory [25]. Here, the pre-stressed configuration is neglected meaning that the free energy function [Eq. (5)] has been built in such a way that it gives zero stress if the artery is not loaded. In this test case, the internal pressure of the blood ($p_{max}$) is assumed to be 25 mmHg (3.3 kPa) that is the typical blood pressure in the pulmonary arteries [14]. It is smaller than the typical systolic pressure, namely 120 mmHg. The reason is that the blood pressure drops as one travels along the vessels of circulatory system and gets farther from the heart.

**Fig. 10:**
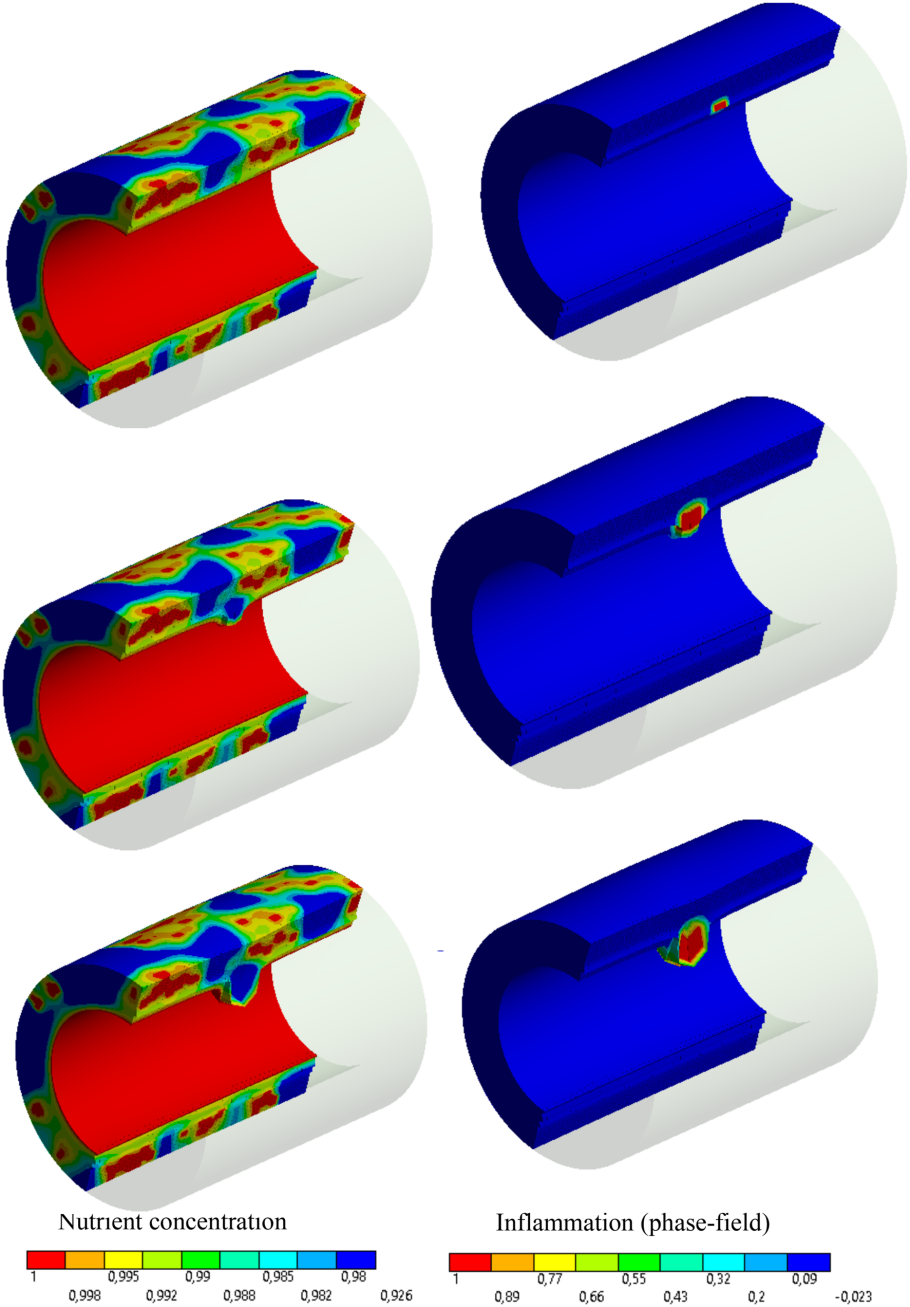
Variation of dimensionless nutrient and inflammation progression in the course of the time (with collagen fiber/with vasa vasorum)

**Fig. 11:**
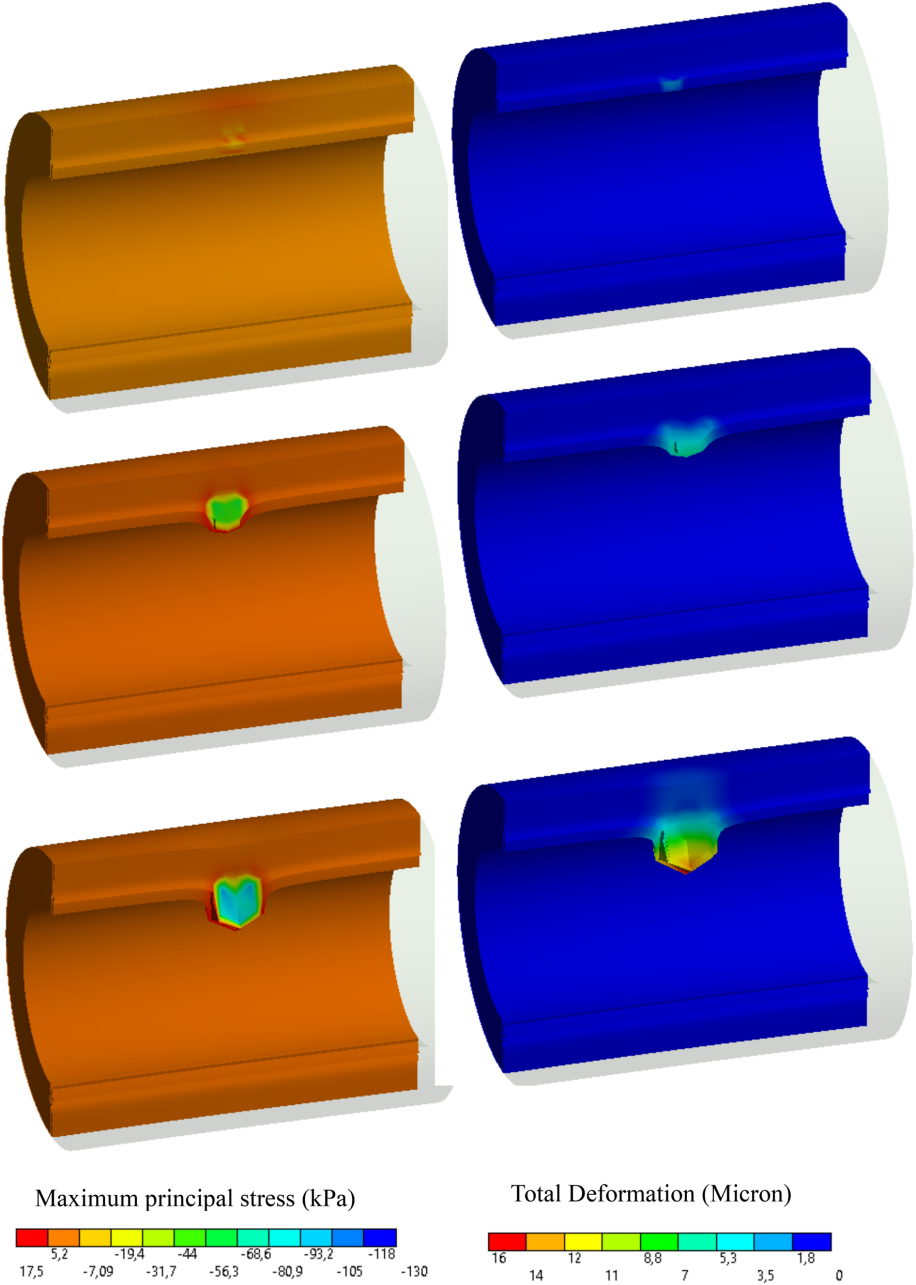
Stress evolution and the deformation of the wall artery due to the inflammation

Similar to 2D cases, Fig. 10 depicts the applicability and robustness of the proposed method in modeling atherosclerosis in a 3D framework. For visualization purposes, only one half of the artery is plotted. As expected, the concentration field reaches its maximum value near the sources (vasa vasorum and the inner surface) and it gradually decays in farther points. The inflammation starts to develop from the initial seed that was introduced artificially in the mid-plane of the artery (see Fig. 4) similar to 2D cases (see Fig. 2). Looking at the stress evolution is also informative in this case. Figure 11 demonstrates the maximum principal stress in the wall and particularly in the proximity of the inflammatory region. One can notice that while the internal regions of the inflammations are under compressive stress due to the overgrowth, the surrounding healthy tissues develop relatively high tensile stresses. It means that a very steep gradient in the stresses is one of the natural characteristics of atherosclerotic arteries. The impact of stress on the growth regulation is not included in this work. It is left for the future work.

A parameter study can be done for all test cases presented here. Such parameter studies are dismissed in this work due to conciseness. The main objective of this paper is to demonstrate the applicability of our mathematical model in simulating the atherosclerosis. Undoubtedly, the prediction capability of such a mathematical model can be improved by performing experimental and clinical investigations. Sparsity and scatteredness of the available experimental data due to the difficulties of in-vitro measurements are the main challenges in this regard, see [15] for example. This is why we have taken approximate and nominal values from the literature [15] for the material parameters in this work without conducting an experimental validation.

#### *Remark*

 It is obvious that one needs to carry out a so-called ”convergence analysis” in order to show that the results are mesh independent. Apart from accuracy that improves upon refining the mesh, some factors dictate the maximum allowable mesh size. It is known that the length scale parameters in the phase field equation (11), namely ($\epsilon$), determines the sharpness of the interface and consequently the maximum allowable element size. Furthermore, the fractal-shaped tree of vasa vasorum necessitates the usage of sufficiently fine mesh in order to capture such small features, see Table 3. Nevertheless, the computational cost increases rapidly if the discretization size decreases . For all 2D and 3D problems presented in this work, the typical mesh size is 0.5 $\upmu{\mathrm{m}}$. We found that this value is, on one hand, small enough to address the the aforementioned concerns and, on the other hand, computationally affordable. Taking into account 4 and 5 degrees of freedom per node in 2D and 3D respectively, it led to a system with approximately 120,000 and 2,000,000 unknowns in 2D and 3D, respectively. It reveals clearly why the 3D cases are substantially expensive compared to 2D ones in terms of computational costs. While the computational running time for 2D cases reaches barely 1 h, the 3D cases need more than 15 hours assuming that an ordinary personal computer (PC) is used.

## Conclusion

A new mathematical model for the simulation of atherosclerosis was proposed and implemented in an FEM framework. The model is based on years of clinical observations during heart and vascular operations by cardiac surgeon Professor Haverich who proposed a new theory on the cause of the atheriosclerosis which differs from the old doctrine of fat deposition (calcification). A multi-field approach consisting of three governing equations was developed to describe mechanical deformation, nutrient transport and biological inflammatory response. The simplifying assumptions and the underlying hypotheses were clarified. The model is capable of capturing atherosclerosis as a result of disruption in the nutrient flow inside the vasa vasorum, the network of micro vessels nourishing the artery. In practice, the occlusion of vasa vasorum may happen due to an inflammatory reactions caused by viruses, bacteria, fine dusts, as well as fat particles (oxidized LDL cholesterol). A clear correlation between an increased heart attack rate and the occurrence of flu epidemics with pneumonia and also exposure to fine dust has been established that support the newly proposed hypothesis for the atherosclerotic plaque. Especially the recent COVID-19 pandemic is of high relevance to this matter, since it does affect the microcirculatory blood vessels in many organs. The involvement of vasa vasorum has clearly been shown by Kawasaki syndrome, an inflammatory disease affection children with COVID 19 infection [6]. Mathematical modeling of atherosclerosis is inevitably necessary in developing any preventative or therapeutic measure against the disease. Both 2D histological examination and 3D full model were provided to demonstrate the robustness, versatility and applicability of the developed numerical tool.

Despite promising results, this work can be improved and extended in a few directions. Firstly, the thermodynamical consistency of the proposed phase-field model should be investigated. In particular, more clinical observations are required about the progression pattern (evolution pattern) of the atherosclerotic plaques. Secondly, a more realistic representation of the artery wall including multiple layers (intima, media, adventia) , several laminae within each layer, elastin, collagen and ground substance can improve the predictiveness of the model. Thirdly, the presented model can be applied to other relevant pathological situation such as arterial “dissection” and “aneurysm”. The Second author believes that the aortic dissection and atherosclerosis are two sides of the same coin and one can hopefully establish a unified theory to explain the pathogenesis based on the vasa vasorum dysfunction, see [22]. As the next possibility and very interesting path for the extension of this work, one can incorporate the impact of stress development into regulating the inflammatory response and the resultant overgrowth. From a mathematical point of view, it results in a two way coupling between the inflammation and stress instead of currently existing connection that is one way. It means that in this work, the inflammation is the “cause” and the deformation and the stress are the “effects” not vise versa. A reciprocal causal relation might be more physical and undoubtedly more challenging. The first author has developed a stress-regulated growth model by means of the principal directions of the stress tensor that can be integrated into this work, see [39]. Finally and needless to say that the computational costs of 3D cases are not comparable to 2D ones. Especially, due to the presence of phase field method, one should be careful about the maximum allowable mesh size for a particular degree of interface sharpness. The narrower the interface is, the finer mesh should be applied and consequently the more expensive the simulation becomes. Hence, extending the numerical tool concerning the “adaptivity” is crucial if one intends to resolve the variable in fine length scales.
